# Facilitating the reduction of V–O bonds on VO_*x*_/ZrO_2_ catalysts for non-oxidative propane dehydrogenation†

**DOI:** 10.1039/d0sc00690d

**Published:** 2020-03-16

**Authors:** Yufei Xie, Ran Luo, Guodong Sun, Sai Chen, Zhi-Jian Zhao, Rentao Mu, Jinlong Gong

**Affiliations:** Key Laboratory for Green Chemical Technology of Ministry of Education, School of Chemical Engineering and Technology, Tianjin University, Collaborative Innovation Center of Chemical Science and Engineering Tianjin 300072 China jlgong@tju.edu.cn

## Abstract

Supported vanadium oxide is a promising catalyst in propane dehydrogenation due to its competitive performance and low cost. Nevertheless, it remains a grand challenge to understand the structure–performance correlation due to the structural complexity of VO_*x*_-based catalysts in a reduced state. This paper describes the structure and catalytic properties of the VO_*x*_/ZrO_2_ catalyst. When using ZrO_2_ as the support, the catalyst shows six times higher turnover frequency (TOF) than using commercial γ-Al_2_O_3_. Combining H_2_-temperature programmed reduction, *in situ* Raman spectroscopy, X-ray photoelectron spectroscopy and theoretical studies, we find that the interaction between VO_*x*_ and ZrO_2_ can facilitate the reduction of V–O bonds, including V=O, V–O–V and V–O–Zr. The promoting effect could be attributed to the formation of low coordinated V species in VO_*x*_/ZrO_2_ which is more active in C–H activation. Our work provides a new insight into understanding the structure–performance correlation in VO_*x*_-based catalysts for non-oxidative propane dehydrogenation.

## Abstract 2

Low coordinated VO_*x*_ species on ZrO_2_ with more reduced V–O bonds leads to improved catalytic activity for propane dehydrogenation.

## Graphical abstract

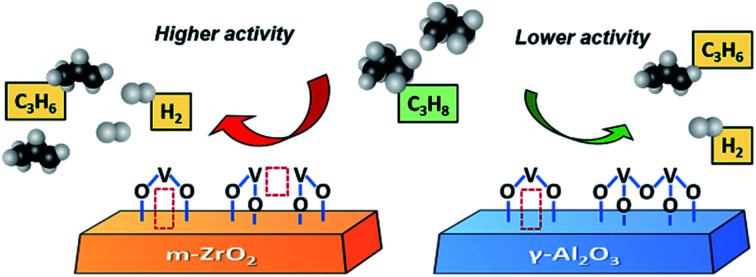

## Introduction

Propylene is one of the most important chemical building blocks and its high-value derivatives are of great demand in the chemical industry. Due to the large-scale exploitation of shale gas, direct propane dehydrogenation becomes a particularly important method to produce propylene using propane as feedstock. Commercialized propane dehydrogenation plants generally utilize Pt-based and CrO_*x*_-based catalysts, however these two kinds of catalysts are either expensive or toxic.^1^ Alternatively, supported vanadium oxide is a promising catalyst compared with Pt and CrO_*x*_ for its competitive performance, low cost and low toxicity.^2,3^

It is well established that VO_*x*_-based catalysts can be utilized in propane oxidative dehydrogenation (ODH).^4–8^ However, there is a trade-off effect between conversion and selectivity associated with over oxidation to CO_*x*_, which prevents the ODH process from achieving high propylene yield. In contrast, better reactivity and selectivity, especially excellent regeneration stability can be achieved when supported VO_*x*_ catalysts are used in the non-oxidative propane dehydrogenation (PDH) process.^9–13^ Nevertheless, the structure–performance correlation of VO_*x*_-based catalysts is still unclear due to the structural complexity of the supported VO_*x*_ catalytic system. Previous studies have confirmed that several factors could influence the performance of VO*_x_*-based catalysts such as polymerization forms of VO_*x*_, chemical states of V and the identity of the support.^14–18^

The support effect is an essential parameter because VO_*x*_ can be bonded to a support through the V–O–support interaction. The variety of supports could lead to significant changes in the catalytic properties of VO_*x*_. It is generally accepted that the support could affect reactivity by stabilizing the active sites and/or altering the electron state of active sites.^19–22^ The support effect has been extensively studied over VO_*x*_-based catalysts in the ODH process. Researchers have concluded that the lattice oxygen in V=O and V–O–support (V–O–S) bonds is consumed in C–H activation and H_2_O is formed. Thus, support identities could affect the reaction by tuning the oxygen vacancy formation energy, which is confirmed by experiments and DFT calculations.^23–25^

However, the support effect works in an entirely different way in the PDH process because not only active sites but also reaction mechanisms are distinct from those of the ODH process. V–O bonds directly catalyze C–H activation forming H_2_ rather than H_2_O. Previous studies found that the reactivity of PDH is dependent on the bond strength of V–O–S.^22^ It is proposed that V–O–S bonds are active sites. In addition, the support identity also influences the behavior of carbon deposition on VO_*x*_ based catalysts, which inversely affects activity and on-stream stability.^12,18^ Nevertheless, the effect of a support is still not clear because the structure of VO_*x*_ and the VO_*x*_–support interface in a reduced state is still ambiguous.

Herein, we explore the support effect on the catalytic performance of VO_*x*_-based catalysts for PDH and provide a new insight into understanding how support identity matters. We discovered that VO_*x*_/ZrO_2_, a well-known catalyst for ODH,^26–28^ has a much more superior PDH performance than commonly used VO_*x*_/Al_2_O_3_. The turnover frequency (TOF) is six times higher by loading VO_*x*_ on ZrO_2_ than on Al_2_O_3_. The remarkable improvement of reactivity has not been reported in previous studies. Rate measurement of catalysts with gradient V loadings was employed to identify the active site of the VO_*x*_/ZrO_2_ catalyst. *In situ* Raman spectroscopic measurements, X-ray photoelectron spectroscopy (XPS) and density functional theory (DFT) calculations were used to determine the structure evolution of VO_*x*_ under a reducing atmosphere, and they show that many more V–O bonds on ZrO_2_ (V=O, V–O–V and V–O–Zr) are consumed during reduction. On this basis, we proposed that the facile reducing nature of V–O bonds promotes the formation of lower coordinated V species which accounts for C–H activation enhancement.

## Results and discussion

### Catalyst structure

**Fig. 1:**
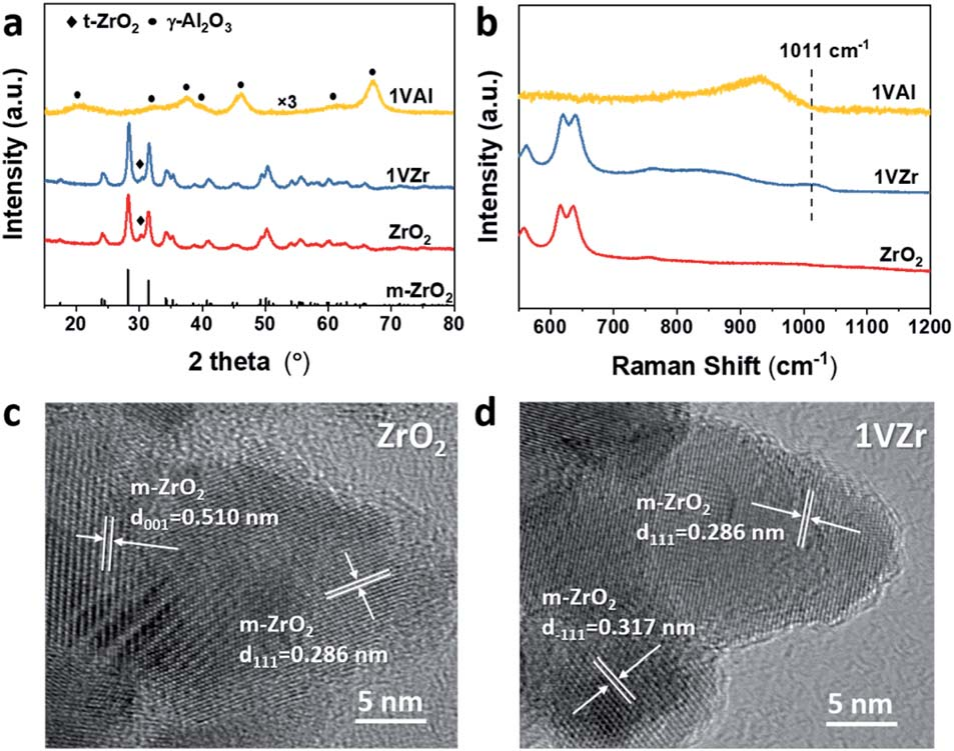
(a) XRD patterns of the ZrO_2_ support and VO_*x*_ supported on ZrO_2_ and Al_2_O_3_. (b) Raman spectra (532 nm excitation) of the ZrO_2_ support and VO_*x*_ supported on ZrO_2_ and Al_2_O_3_. TEM images of (c) ZrO_2_ and (d) VO_*x*_/ZrO_2_.

A series of characterization techniques were used to determine the bulk and surface structure of the catalysts (VO_*x*_ loaded on ZrO_2_ and Al_2_O_3_ are denoted as *x*VZr and *x*VAl where *x* represents the mass percentage of V, metal base). The bare ZrO_2_ support, 1VZr and 1VAl were characterized by X-ray diffraction (XRD). As shown in Fig. 1a, ZrO_2_ is mainly composed of monoclinic phase (m-ZrO_2_, JCPDS 72-1669) with small amount of a tetragonal phase (t-ZrO_2_, JCPDS 79-1771), which is consistent with the previous report using the same preparation method.^29^ The same XRD pattern of 1VZr and pure ZrO_2_ indicates that VO_*x*_ does not change the crystalline structure of ZrO_2_. In addition, for VO_*x*_ loaded catalysts, only diffraction lines of the support could be detected (Fig. 1a and S1a†), which means amorphous VO_*x*_ is well dispersed on these catalysts.^30,31^

Vis-Raman spectroscopic measurements, which are sensitive to the presence of V_2_O_5_, were performed over the catalysts to gain insight into the kind of vanadium species. Raman spectra of the ZrO_2_ support, 1VZr and 1VAl are displayed in Fig. 1b. A band at 1011 cm^−1^ is attributed to vanadyl stretching of surface-dispersed VO_*x*_. No sharp band at 995 cm^−1^, assigned to the stretching vibration of V=O in crystal V_2_O_5_, is detected. With the V loading increasing, a band characteristic of V_2_O_5_ at 995 cm^−1^ appears after the loading reached 3 wt% (Fig. S1b†). This demonstrates the existence of crystal V_2_O_5_ in 3VZr and 4VZr. From the enlarged region on the left from 75 to 160 cm^−1^, a small band which corresponds to V_2_O_5_ at around 150 cm^−1^ can be seen in 2.5VZr. This implies that VO_*x*_ begins to crystalize at 2.5VZr. Besides, the absence of a peak at around 770 cm^−1^ suggests no ZrV_2_O_7_ is formed in our samples.^32^ The Raman spectra demonstrate that VO_*x*_ species are well dispersed as oligomeric species when the loading is less than 2 wt% and V_2_O_5_ crystals are formed after the loading reaches 2.5 wt%.

Surface density of V was calculated through the BET surface area and V loading. The detailed results are listed in Table S1.† 2.5VZr has a V density of 6.9 nm^−2^ which is a bit lower than the theoretical monolayer coverage. It has been proved that the presence of V_2_O_5_ is inevitable below theoretical monolayer coverage by a simple incipient wetness impregnation method.^10^ This agrees with the observation of the V_2_O_5_ peak in 2.5VZr through Raman spectroscopic measurements.

The morphologies of ZrO_2_ and 1VZr were also characterized by TEM (Fig. 1c and d). ZrO_2_ is in the form of a nanoparticle with a relevant uniform size of around 30 nm and its morphology does not change after loaded with VO_*x*_. Lattice fringes of V_2_O_5_ could not be found because amorphous VO_*x*_ is well dispersed on the surface of ZrO_2_. The TEM results are in good agreement with the observations of XRD and Raman results.

### Catalytic performance

**Fig. 2:**
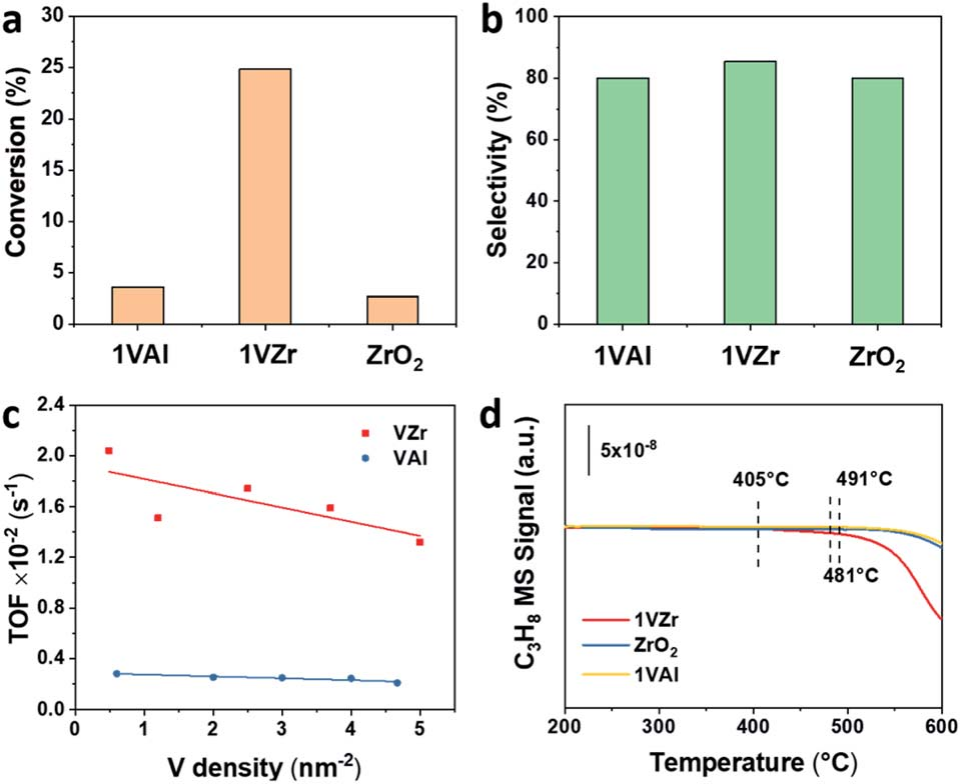
(a) Initial propane conversion and (b) propylene selectivity (based on all products) of 1VAl, 1VZr and ZrO_2_. Reaction conditions: *m*_cat_ = 0.4 g; C_3_H_8_ : N_2_ : H_2_ = 7 : 36 : 7; *T* = 550 °C; inlet flow = 50 mL min^−1^. (c) Comparison of TOF values between VZr and VAl with different V densities. (d) C_3_H_8_-TPSR of 1VZr, ZrO_2_ and 1VAl.

Catalytic performance of VO_*x*_ supported on ZrO_2_ and γ-Al_2_O_3_ for the PDH reaction was studied. The initial propane conversion and propylene selectivity based on all products are illustrated in Fig. 2a and b. The initial conversion of 1VZr is approximately five times higher than that of 1VAl and pure ZrO_2_, which shows a significant support effect. The total selectivity towards propylene is around 80% and gas-phase selectivity towards it is >90% (Fig. S2a†) for all catalysts, which are at a similar level with respect to those of the VO_*x*_-based catalysts in previous studies.^10,14,18,33^ In addition, we performed 120 min of the on stream reaction and studied the reaction–regeneration cycles over 1VZr (Fig. S2b and S3†). The propane conversion and propylene selectivity exhibit little change during six cycles, implying outstanding regeneration stability.

Because the surface areas of ZrO_2_ and Al_2_O_3_ are different, when the V loadings of VZr and VAl are identical, they have different V densities. Considering the activity is also influenced by V density, the TOFs of VZr and VAl were also compared based on V density (Fig. 2c and Table S2†). The calculated TOF of VZr is almost 6-fold higher than that of VAl and only slightly decreases with the V surface density, which implies that the activity is strongly support-dependent. This phenomenon is similar to that of the GaO_*x*_ catalytic system, where the activity is not a consequence of Ga nuclearities, but of the Ga–O–support interaction.^34^

To further evaluate the intrinsic activity in C–H activation, propane temperature-programmed surface reaction (C_3_H_8_-TPSR) experiments were carried out over 1VZr, ZrO_2_ and 1VAl. The temperature where the C_3_H_8_ signal begins to drop is determined as the C–H activation temperature. As shown in Fig. 2d, the initial C–H activation temperature of 1VZr is around 405 °C, which is almost 80 and 90 °C lower than that of ZrO_2_ (481 °C) and 1VAl (491 °C). Moreover, the C–H activation temperature of 3VAl (similar V density to 1VZr, Fig. S4†) was also tested, which is 100 °C higher than that of 1VZr. Along with the TOF difference between VZr and VAl, we can conclude that the intrinsic C–H activation ability of 1VZr is distinguishable.

### Active phase identification

It has been proved that pure ZrO_2_ can also catalyze propane dehydrogenation through coordinatively unsaturated Zr (Zr_cus_).^35–37^ In addition, Jeon *et al.* prepared V–Zr mix oxide and found that V incorporation into the ZrO_2_ bulk phase would promote generation of more Zr_cus_.^38^ Although in our VO_*x*_/ZrO_2_ system, VO_*x*_ is supported on the surface of a support, not in the bulk phase, there still remains different possibilities for this superior performance of the VO_*x*_/ZrO_2_ catalyst: ZrO_2_ enhances the activity of VO_*x*_, VO_*x*_ enhances the activity of ZrO_2_ (Zr_cus_), or a combination of the two. Therefore, it is essential to verify if the active phase is VO_*x*_ or Zr_cus_ in order to understand the origin of this dramatic performance improvement. To this end, various experiments were performed to figure out whether the catalytic performance is due to VO_*x*_ or Zr_cus_ or both of them.

**Fig. 3:**
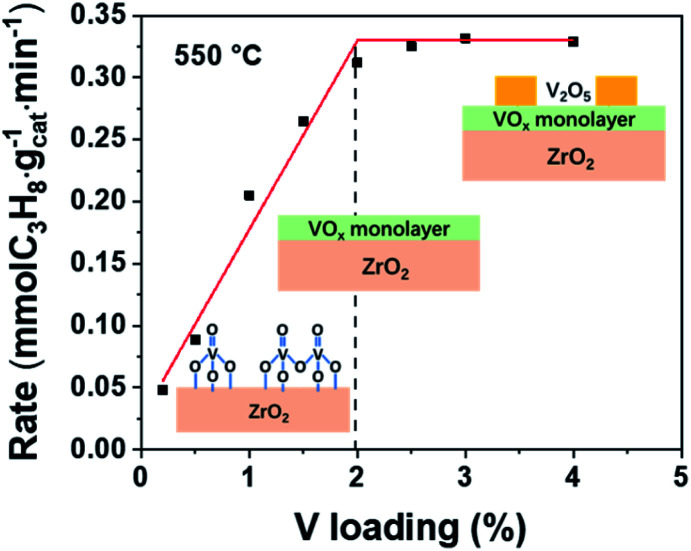
C_3_H_8_ reaction rate *versus* different V loadings on VZr catalysts at 550 °C.

**Fig. 4:**
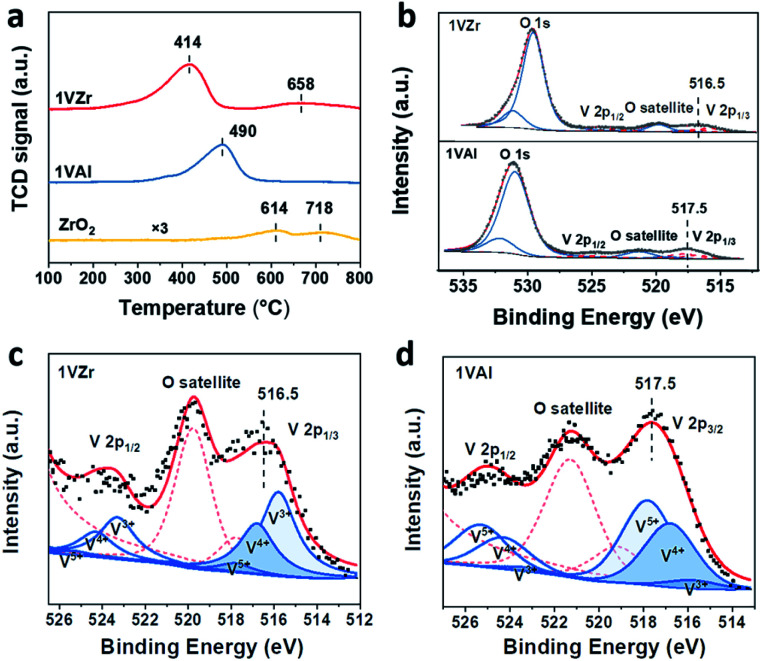
(a) H_2_-TPR profiles of 1VZr, 1VAl and pure ZrO_2_. (b) XPS O 1s and V 2p peaks for 1VZr and 1VAl. Deconvolution results of V 2p_3/2_ for (c) 1VZr and (d) 1VAl after reduction for 30 minutes.

The propane consumption rate *versus* V loading is shown in Fig. 3. The reaction rate rises linearly as the V loading increases until the V loading reaches 2 wt%, indicating VO_*x*_ should be the active component. After this linearly increasing period, the C_3_H_8_ consumption rate does not change with increasing V loading because full VO_*x*_ overlayers are formed at 2 wt% and crystalized V_2_O_5_ appears afterward as confirmed by the Raman spectra (Fig. S1b†). In addition, previous articles reported a shift of XPS Zr 3d peaks and reduction peaks of ZrO_2_ seen in the H_2_ temperature programmed reduction (H_2_-TPR) profile if Zr_cus_ is the active site.^37,39,40^ However, these phenomena cannot be observed in the VO_*x*_/ZrO_2_ system. No ZrO_2_ reduction peak can be observed up to 600 °C (Fig. 4a), while our reactions were conducted at 550 °C. In addition, the XPS test also confirms that the binding energy of Zr 3d in ZrO_2_ and 1VZr has no difference (Fig. S5†). These results provide evidence that ZrO_2_ is not the active phase in VZr catalysts. Along with catalytic activity being linearly related to the amount of V, which directly proves VO_*x*_ is responsible for the activity, we suggest VO_*x*_ rather than Zr_cus_ to be the main active phase. However, it is a pity that these experiments cannot identify which kind of V–O bond (V=O, V–O–V, V–O–Zr or all of them) is responsible for C–H activation. A potential method to solve this problem is Surface Organometallic Chemistry (SOMC),^41–43^ which provides access to producing well-defined isolated VO_*x*_ only possessing V=O and V–O–support. This direction still needs further investigation.

### Structure–performance correlation

H_2_-TPR experiments were performed to test the reducibility and VO_*x*_–support interaction of the catalysts (Fig. 4a). The main peak at around 400–500 °C is attributed to the reduction of VO_*x*_ and peaks above 600 °C are ascribed to a partial reduction of ZrO_2_.^36,44–46^ The reduction temperature of VO_*x*_ for 1VZr is lower than that for 1VAl. Considering that the reduction temperature is influenced by the dispersion of VO_*x*_,^15,47^ we performed H_2_-TPR on a series of VAl and VZr catalysts (Fig. S6†). The reduction temperatures of VZr catalysts are at about 400 °C while those of VAl catalysts are at about 500 °C. Thus, the low reduction temperature of the VZr catalyst implies that the interaction between VO_*x*_ and ZrO_2_ is weaker than that between VO_*x*_ and Al_2_O_3_. This phenomenon is further discussed in the *in situ* Raman spectroscopy section below. Alternatively, the peak area of 1VZr is larger than that of 1VAl. We calculated the H : V ratio through the peak area and estimated the average oxidation state (AOS) of V. As listed in Table S3,† the AOS of V in 1VZr and 1VAl corresponds to 3.5 and 4.0, which indicates that VO_*x*_ can be more readily and deeply reduced on ZrO_2_.

To further identify the valance state of V, XPS investigation was carried out over 1VZr and 1VAl after H_2_ reduction for 30 min. V 2p core levels of 1VZr and 1VAl are displayed in Fig. 4b. We compare the binding energy of V in different catalysts qualitatively. The binding energy of V 2p_2/3_ centers at 516.5 eV in 1VZr and 517.5 eV in 1VAl. This indicates that the valance state of V in 1VZr is lower than that in 1VAl. According to the deconvolution results of V 2p shown in Fig. 4c, d and Table S4,† the oxidation state of V is a mixture of V^5+^, V^4+^ and V^3+^, which is consistent with previous results.^33,44,48^ The fraction of V^3+^ is 48.6% in 1VZr, which is higher than 6.6% in 1VAl.

It has been elucidated in the former articles that V^3+^ is more active in the PDH reaction.^14,33^ However, seldom detailed analyzes have been conducted to identify the structure of VO_*x*_ in a reduced state and the structure–performance correlation is not clear. Therefore, *in situ* UV-Raman spectroscopy, which is more sensitive to the surface VO_*x*_ structure,^49^ was performed to monitor the evolution of V=O, V–O–V, and the V–O–support.

**Fig. 5:**
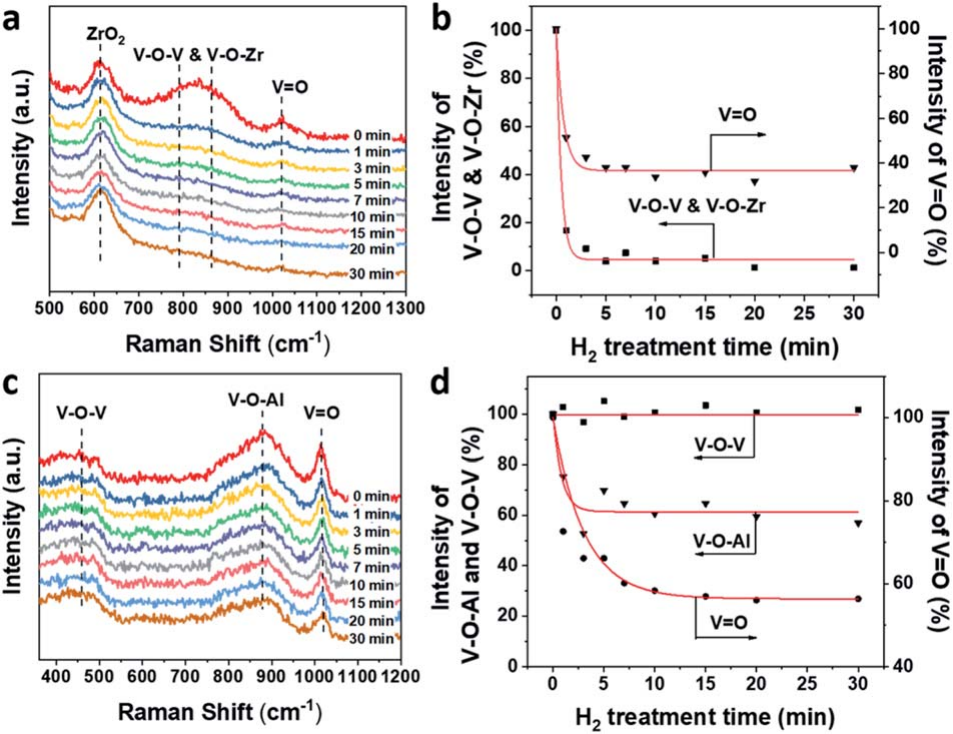
(a) *In situ* UV-Raman (325 nm excitation) spectra of VZr during H_2_ reduction at 550 °C. (b) Intensities of V–O–V, V–O–Zr, and V=O *versus* H_2_ treatment time. (c) *In situ* UV-Raman (325 nm excitation) spectra of 1VAl during H_2_ reduction at 550 °C. (d) Intensities of V–O–V, V–O–Al and V=O *versus* H_2_ treatment time.

The evolution of V–O–V, V–O–Zr and V=O in 1VZr during H_2_ reduction is shown in Fig. 5a and b. A broad band at 750–950 cm^−1^ is ascribed to V–O–V and V–O–Zr bonds.^32,50^ The band at around 1020 cm^−1^ is assigned to V=O in dispersed VO_*x*_.^27,32,51^ The number of V–O–V and V–O–Zr bonds decreases substantially which implies that plenty of these bonds are consumed during reduction. In addition, nearly 60% of V=O in 1VZr disappeared after reduction for 30 min. The comparative experiment of 1VAl is displayed in Fig. 5c and d. The bands attributed to V–O–V and V–O–Al on the Al_2_O_3_ support are located at 400–600 cm^−1^ and 910 cm^−1^ respectively.^49^ A sharp band at 1018 cm^−1^ is ascribed to V=O.^49^ In contrast with V–O–Zr and V–O–V in the 1VZr sample, the V–O–Al band only decreases to 60% of that for fresh 1VZr and no change of the V–O–V band is detected which implies V–O–V bonds on the Al_2_O_3_ support cannot be reduced under H_2_ treatment. Meanwhile, 40% of V=O in 1VAl is reduced, which is less than that in 1VZr. The result of *in situ* Raman spectroscopy confirms that not only V=O and V–O–Zr, but also V–O–V can be deeply reduced in 1VZr which results in the deeper reduction degree of 1VZr observed in H_2_-TPR and XPS studies.

The facile reduction nature of V–O bonds in the VZr catalyst can be interpreted by the lower electronegativity of Zr compared with Al, which causes the difference of the interaction between VO_*x*_ and the support. It has been shown that a lower support cation electronegativity can result in a higher electron density of the V–O–S bond, which lead to these bonds being readily reduced.^52,53^ Along with the lower reduction temperature of VZr shown in the H_2_-TPR profile, it can be deduced that the interaction between VO_*x*_ and ZrO_2_ weakens the V–O bonds thus resulting in the facile reduction nature of the VZr catalyst.

Note that when O atoms are removed during the reduction period, the chemical environment of V changes, which leads to the formation of coordinatively unsaturated V. It is established that metal cations (Ga^3+^, Zn^2+^, Zr^4+^, *etc.*) with a low coordination number are more active for the reaction.^54^ As more V–O bonds are reduced in the VZr catalyst, we could deduce that lower coordinated V sites are more active for the C–H activation. Our recent DFT calculations also proved that coordinatively unsaturated VO_*x*_ was more active in C–H activation^55^ while this work gives experimental evidence.

### DFT calculations

DFT calculations were conducted to gain further insight into the structure of the active site and its influence on catalytic performance. Noting that the TOF difference between VZr and VAl exists with all the V densities, we constructed dimeric vanadium oxide species on the m-ZrO_2_(1̄11) and γ-Al_2_O_3_(100) surface as representatives^36,55^ (Fig. S7†).

We calculated oxygen vacancy formation energy of different oxygen atoms, with the energy of H_2_O(g) and H_2_(g) as the reference, in these vanadium oxide dimers to confirm the different reducibilities they showed in our experiment results. As listed in Table 1, oxygen atoms are removed from V=O, V–O–V and the V–O–support. Four of five V–O bonds in VZr (V=O, V–O–V and two V–O–Zr) have relatively low oxygen formation energies while only two of five in VZr do (V=O, V–O–Al). The calculated oxygen vacancy formation energies are in good agreement with the Raman spectroscopy result that V=O, V–O–V and V–O–Zr bonds in VZr can be reduced under a H_2_ atmosphere while only V=O and part of V–O–Al are reduced in VAl.

**Table 1:** Oxygen vacancy formation energies of vanadium supported on m-ZrO_2_(1̄11) and γ-Al_2_O_3_(100)

	Oxygen vacancy formation energies (eV)				
	V=O	V–O–V	V–O–support		
V_2_O_5_/m-ZrO_2_(1̄11)	0.13	−0.13	0.03	0.14	0.34
V_2_O_5_/γ-Al_2_O_3_(100)	−0.07	0.44	0.36	0.35	−0.25

**Fig. 6:**
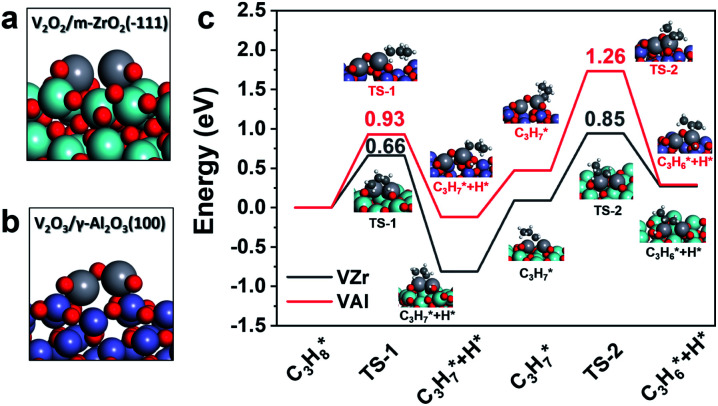
Reduced catalyst models of (a) V_2_O_2_/m-ZrO_2_(1̄11) and (b) V_2_O_3_/γ-Al_2_O_3_(100). (c) Calculated potential energy diagrams of the first and second propane dehydrogenation step.

Since VZr and VAl have different degrees of reduction, we constructed V_2_O_2_/m-ZrO_2_(1̄11) and V_2_O_3_/γ-Al_2_O_3_(100) models to represent the catalyst structure in a reduced state (Fig. 6a and b). In terms of the *in situ* Raman results and calculated oxygen vacancy formation energies, three (one in each V=O, V–O–Zr and V–O–V) and two (one V=O and one V–O–Al) V–O bonds with the lowest oxygen vacancy formation energies were removed from the initial dimeric V_2_O_5_ structure respectively. The propane dehydrogenation barriers over partially reduced VZr and VAl were computed and transition state (TS) geometries are shown in Fig. 6c. The first and second step C–H activation barriers in VZr is 0.66 eV and 0.85 eV, respectively, while in VAl they are 0.99 eV and 1.26 eV, respectively. DFT calculations also confirm that low coordinated V species are more active in C–H activation, which is consistent with the highest TOF and the lowest C–H activation temperature for VZr observed in the catalytic performance and C_3_H_8_-TPSR tests.

**Scheme 1:**
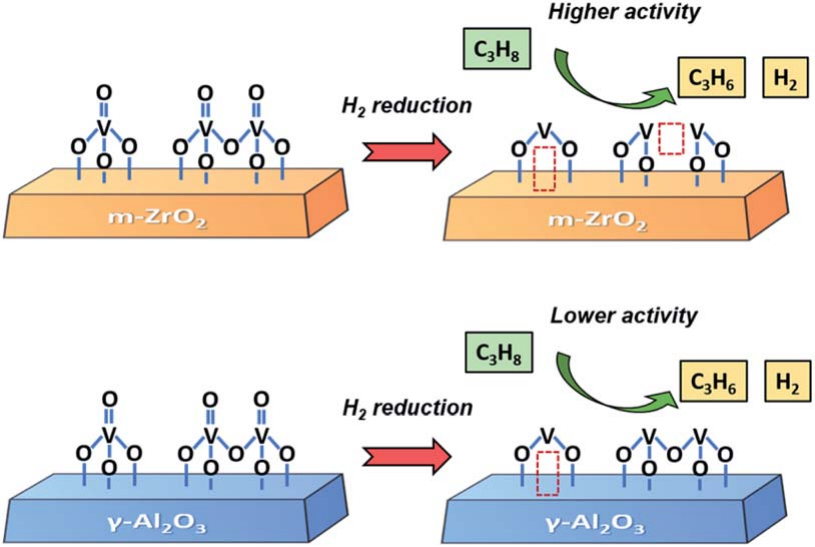
Propane dehydrogenation on VZr and VAl catalysts with different V structures and VO_*x*_ reduction degrees.

Based on the experimental and theoretical results, we propose a structure–performance correlation of VZr and VAl catalysts, as shown in Scheme 1. VO_*x*_ can be readily reduced on ZrO_2_ because the interaction between VO_*x*_ and ZrO_2_ facilitates the reduction of V=O, V–O–V and V–O–Zr bonds. However, for the VAl catalyst, only V=O and some V–O–Al bonds can be reduced. Thus, more low coordinated V species form in the VZr catalyst during reduction, and these low coordinated V species exhibit a better performance in PDH.

## Conclusions

In summary, we prepared VO_*x*_ loaded on ZrO_2_ through a simple incipient wetness impregnation method, which exhibits a dramatically improved performance compared with VO_*x*_ loaded on Al_2_O_3_ for propane dehydrogenation. The TOF_C_3_H_8__ of 1VZr is determined to be 0.0161 s^−1^, which is almost six times higher than that of 1VAl.

We further prove that the remarkable reactivity of the 1VZr catalyst was attributed to the promotion of C–H activation over VO_*x*_ species rather than it over ZrO_2_. Besides, combining *in situ* Raman and XPS spectroscopy results, we propose the enhanced C–H activation on VZr results from the facile reduction of V=O, V–O–V and V–O–Zr bonds, thus producing deeply reduced and lower coordinated V species. DFT calculations also confirm that the C–H rupture energy barrier is lower for partially reduced VZr with low coordinated V species. Considering the dramatic performance achieved through the interaction between VO_*x*_ and ZrO_2_, our work provides a new insight into high-performance VO_*x*_-based catalysts for propane dehydrogenation.

## Conflicts of interest

There are no conflicts to declare.

## Supplementary Material

SC-011-D0SC00690D-s001
