# Supplementary material for: Facilitating the reduction of V–O bonds on VOx/ZrO2 catalysts for non-oxidative propane dehydrogenation
Source: Chem Sci. 2020 Mar 16;11(15):3845–51. doi: 10.1039/d0sc00690d (PMC8152552; doi:10.1039/d0sc00690d)
Supplement: SC-011-D0SC00690D-s001 [file SC-011-D0SC00690D-s001.pdf]

Electronic Supplementary Material (ESI) for Chemical Science.
This journal is © The Royal Society of Chemistry 2020

Electronic Supporting Information

Facilitating the Reduction of V-O Bonds on VO_x/ZrO_2 Catalysts for Non-oxidative Propane Dehydrogenation

Yufei Xie, Ran Luo, Guodong Sun, Sai Chen, Zhi-Jian Zhao, Rentao Mu, and Jinlong Gong*

Key Laboratory for Green Chemical Technology of Ministry of Education, School of Chemical Engineering and Technology, Tianjin University; Collaborative Innovation Center of Chemical Science and Engineering, Tianjin 300072, China;

*Corresponding authors: jlgong@tju.edu.cn

Contents

S1. Experimental and computational methods.....2
S2. Supporting tables and figures.....4
Reference.....17

S1. Experimental and computational methods

1.1 Characterization methods

X-ray diffraction (XRD) measurements were performed on a Rigaku C/max-2500 diffractometer with CuKα radiation. Raman measurements of VZr with different loadings were conducted under ambient condition on a Renishaw inVia reflex Raman spectrometer equipped with visible (532 nm) Ar-ion laser beam. The samples were dried at 300 °C for 2 h before the measurement.

The measurement of specific surface area (SSA) of the samples was conducted on a Micromeritics Tristar 3000 analyzer at −196 °C. The Brunauer-Emmett-Teller (BET) method was applied to calculate SAAs on the basis of the N_2 isotherms.

H_2-Temperature Program Reduction (H_2-TPR) tests were executed on a Micromeritics AutoChem 2920 apparatus. The sample (0.4 g) was purged at 300 °C for one hour under an Ar stream (20 mL/min). After cooling down to 100 °C, H_2-TPR was conducted in 10 vol % H_2/Ar (30 mL/min) flow. The sample was headed up to 800 °C with a heating rate of 10 °C/min. The signal was detected with a thermal conductivity detector (TCD).

In situ Raman was performed on a Renishaw inVia reflex Raman spectrometer with 325 nm Ar-ion laser beam. The sample was pretreated with 1% O_2/N_2 at 550 °C for 1 hour and recorded the signal. Then, the gas was changed to 10% H_2/Ar and collected the spectra every minute.

XPS tests were executed on a PHI 1600 ESCA instrument (PE Company) equipped with an Al Kα X-ray radiation source (*hv* = 1486.6 eV). Before the test, reduced samples were pretreated under H_2 atmosphere at 550 °C for 30 min. The binding energies were referenced to the C 1s peak at 284.6 eV.

1.2 Reactivity test

Catalytic performance evaluation was carried out in a quartz fixed-bed reactor (8 mm ID) under 0.13 MPa. 0.4 g catalyst mixed with quartz sands were packed into the tube. The sample was heated up to 550 °C under N_2 flow (36 mL/min) and then reduced at the same temperature under H_2 atmosphere (H_2:N_2=7:36) for 30 minutes. Afterward, a mixture of C_3H_8, N_2 and H_2 (C_3H_8:N_2:H_2=7:36:7) was fed to the reactor at a rate of 50 mL/min. The products were analyzed with an online GC (2060) equipped with a flame ionization detector (Chromosorb 102 column) and a thermal conductivity detector (Al_2O_3 Plot column). Propane conversion and propylene selectivity based on all products and gas-phase products were calculated from eq(1), eq(2) and eq(3).

$$\text{Conversion (X)} = \frac{F_{C_3H_8,in} - F_{C_3H_8,out}}{F_{C_3H_8,in}} \times 100\% \qquad (1)$$

$$\text{Selectivity } (S_{total}) = \frac{F_{C_3H_6,out}}{F_{C_3H_8,in} - F_{C_3H_8,out}} \times 100\% \qquad (2)$$

$$\text{Selectivity } \left(S_{gas\ phase}\right) = \frac{3 \times F_{C_3H_6,out}}{\sum(n_i \times F_{i,out})} \times 100\% \qquad (3)$$

Where i stands for different carbon product in the gas phase. n_i stands for the number of carbon atom in the molecular. F_i stands for the molar flow rate of species i.

Turnover frequency (TOF) and propane conversion rates were determined under a special condition. Total flow rate was determined to eliminate the mass transfer and the conversion below 15% to ensure differential reaction. TOF was calculated based on the total number of V atom from eq(4).

$$TOF_{C_3H_8} = \frac{\left(F_{C_3H_8,in} - F_{C_3H_8,out}\right) \times N_A}{\text{total number of V atoms}} \qquad (4)$$

As VO_x could be well dispersed on ZrO_2 as a monolayer, TOF based on the number of V atom will be same as

TOF based on active sites. This calculation method has been widely applied in many other works[1-6]. Nevertheless, for the catalysts with crystal V_2O_5, the calculated TOF would be smaller than the actual TOF based on the active sites.

1.3 Models and computational details

Both VZr and VAl models and their correspond reduced models were created to investigate reducibility and propane dehydrogenation energy barriers. Monoclinic ZrO_2 unit cell was cut along the $(\bar{1}11)$ plane and γ-Al_2O_3 unit cell along the (100) plane to simulate surfaces of different supports. Results reported in this work use a p(2 x 2) m-ZrO_2 surface unit cell with four layers and p(1 x 2) γ-Al_2O_3 surface unit cell with two Al-O-Al layers. The Brillouin zone was sampled using 2 x 2 x 1 k-points grid for VZr and 3 x 3 x 1 k-points grid for VAl. V_2O_5 clusters with similar structures were added on these surfaces to represent VO_x species. Three characteristic structures aimed to be compared were involved in these clusters: V=O, V-O-V and V-O-support.

Calculations were performed using Vienna ab initio simulation package (VASP)[7, 8]. In order to correct on site Coulomb correlation of occupied V 3d orbitals, we employed the gradient-corrected exchange−correlation functional by Perdew, Burke, and Ernzerhof (PBE)[9] and an effective Hubbard-type U parameter of 3.2 eV. The valence wave functions were expanded by plane wave with a cutoff energy of 400 eV. The atomic core was described by the Projected Augmented Wave (PAW) pseudopotentials[10].

Due to the overbinding of GGA in the O_2 molecule, we used H_2(g) and H_2O(g) as reference for oxygen vacancy formation energy:

$$\Delta E_V = E_{\text{surface with Ov}} + E_{H_2O(g)} - E_{\text{clean surface}} - E_{H_2(g)} \qquad (5)$$

In potential energy diagrams, the energy of C_3H_8 in the gas phase is taken as reference and the energy of intermediates are corrected with H_2 in the gas phase. The adsorption energy is defined as:

$$\Delta E_{\text{ads}} = E_{C_3H_x\text{+surface}} + \left[\frac{8-x}{2}\right] E_{H_2(g)} - E_{\text{clean surface}} - E_{C_3H_8(g)} \qquad (6)$$

Moreover, the transition states were located by the climbing-image nudged elastic band method (NEB)[11]. The activation barrier E_a was calculated based on following equation:

$$\Delta E_a = E_{\text{transition state}} - E_{\text{initial state}} \qquad (7)$$

S2. Supporting tables and figures

2.1 Supporting tables

Table S1. Catalysts and their V loadings (ω(V)), BET surface areas (S_{BET}), V surface densities and dispersion states.

Sample	V loading (wt.%)	BET surface area (m^2/g)	V density (nm^{-1})	Type of VO_x formed*
0.2VZr	0.2	48.6	0.49	Sub-monolayer
0.5VZr	0.5	50.7	1.2	Sub-monolayer
1VZr	1	48.2	2.5	Sub-monolayer
1.5VZr	1.5	47.9	3.7	Sub-monolayer
2VZr	2	47.0	5.0	Actual monolayer without V_2O_5
2.5VZr	2.5	42.9	6.9	Theoretical monolayer. Little V_2O_5
3VZr	3	42.4	8.4	Monolayer VO_x & V_2O_5
4VZr	4	40.6	11.6	Monolayer VO_x & V_2O_5

*Type of VO_x formed is determined by vis-Raman spectra.

Table S2. Catalysts and their V surface densities, initial rates of C_3H_8 consumption ($r(C_3H_8)$) and TOF values.

Sample	V surface density /nm^{-2}	$r(C_3H_8)$ / $mmol\cdot g^{-1}\cdot min^{-1}$	*TOF /s^{-1}
ZrO_2	—	0.03	—
0.2VZr	0.49	0.048	0.0204
0.5VZr	1.2	0.089	0.0151
1VZr	2.5	0.205	0.0174
1.5VZr	3.7	0.265	0.0159
2VZr	5.0	0.312	0.0132
1VAl	0.6	0.032	0.0025
6VAl	4.0	0.121	0.0017

* TOF is calculated based on the number of V atoms.

Table S3. Numbers of H atoms consumption per V atom and average oxidation state of V.

Sample	H: V*	AOS of V*
1VZr	1.5	3.5
1VAl	1.0	4.0
ZrO_2	-	-

*Calculated from H_2-TPR result.

Table S4. XPS results and deconvolution results of V $2p_{3/2}$.

Sample	BE of lattice oxygen O_I (eV)	BE of adsorbed oxygen O_{II} (eV)	V^{3+}		V^{4+}		V^{5+}	
			BE (eV)	Area (%)	BE (eV)	Area (%)	BE (eV)	Area (%)
1VAl	531.1	532.3	515.8	6.2	516.8	40.1	517.8	53.7
1VZr	529.6	531.2	515.8	58.2	516.8	35.3	517.8	6.5

Table S5. Comparation of catalytic performance in this work with previous works.

Catalysts	Temperature (°C)	WHSV (h^{-1})	TOF (h^{-1})	References
1VZr	550	2.07	57.6	This work
1VAl	550	2.07	9.6	This work
isolated Ga/SiO_2	550	0.33	20.0	Chem. Sci., 2017, 8, 2661-2666
Ga(i-Bu)$_3$/Al_2O_3	550	1.48	27.2*	ACS Catal. 2018, 8, 7566−7577
Ga(i-Bu)$_3$/SiO_2	550	2.36	11.2*	ACS Catal. 2018, 8, 7566−7577
islated Fe/SiO_2	650	0.39	1.4	ACS Catal. 2015, 5, 3494−3503
isolated Cr/SiO_2	550		10.3	Inorg. Chem. 2015, 54, 11, 5065-5078
isolated Cr/Al_2O_3	550		60.0	Organometallics 2017, 36, 1, 234-244
isolated Co/SiO_2	550	0.076	12.6	J. Catal. 2015, 322, 24-37
isolated V/SiO_2	500	0.12	1.66*	Organometallics 2013, 32, 21, 6452-6460

* TOF calculated from given data.

2.2 Supporting figures

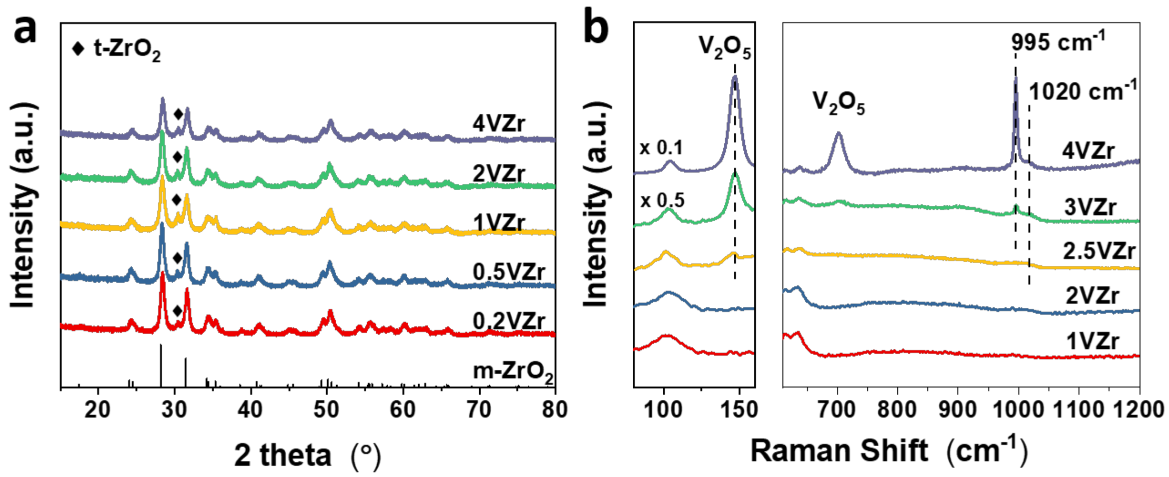

Figure S1. (a) XRD patterns and (b) vis-Raman spectra of VZr catalysts with different V loadings.

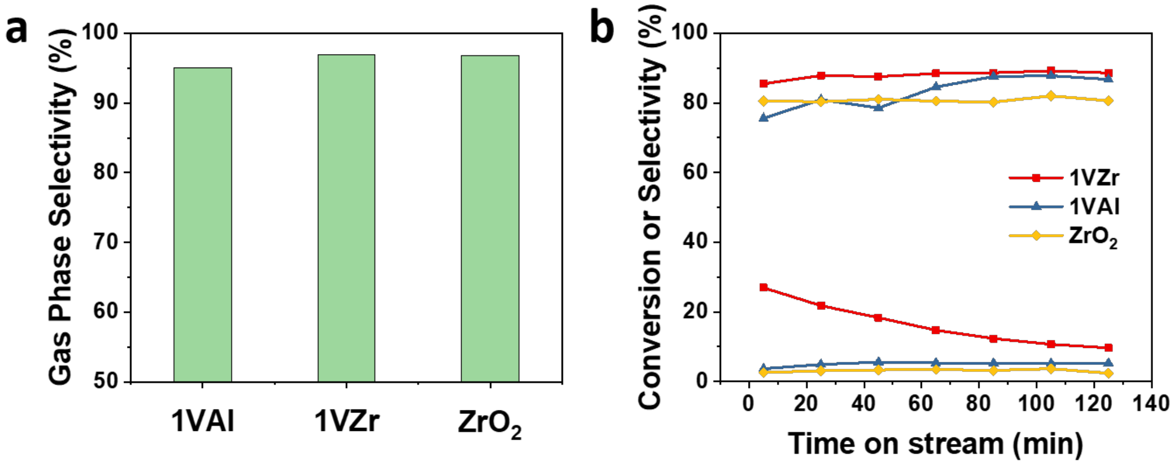

Figure S2. (a) Gas phase selectivity of 1VAl, 1VZr and ZrO_2. Reaction condition: m_{cat}=0.4 g; C_3H_8:N_2:H_2 = 7:36:7; T = 550 °C; inlet flow = 50 mL/min. (b) Propane conversion and propene total selectivity of 1VZr, 1VAl and ZrO_2.

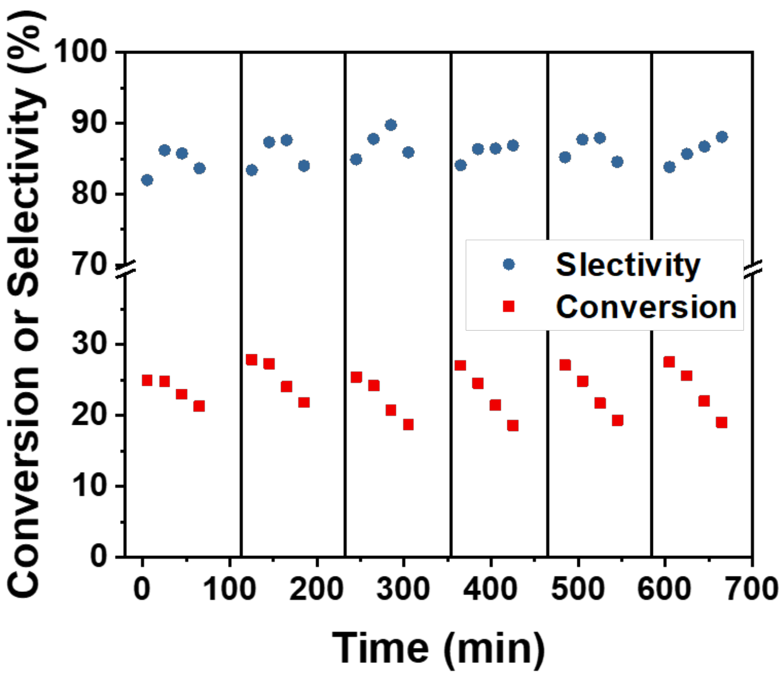

Figure S3. Propane conversion and selectivity of 1VZr during reaction-regeneration cycles. Reaction condition: m_{cat} = 0.4 g; C_3H_8:N_2:H_2 = 7:36:7; T = 550 °C; inlet flow = 50 mL/min

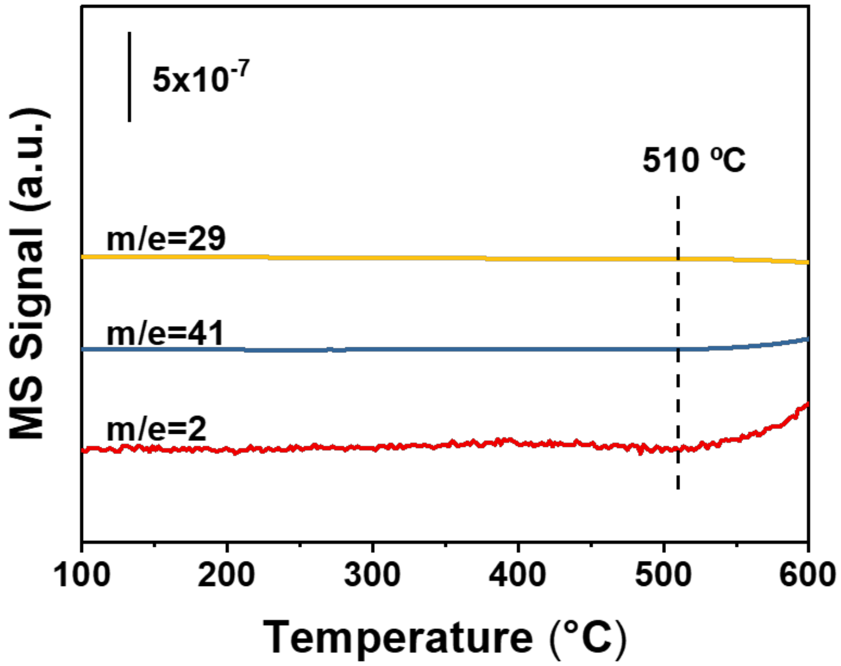

Figure S4. C_3H_8-TPSR of 3VAl. Propane: m/e = 29. Propene: m/e = 41. H_2: m/e = 2.

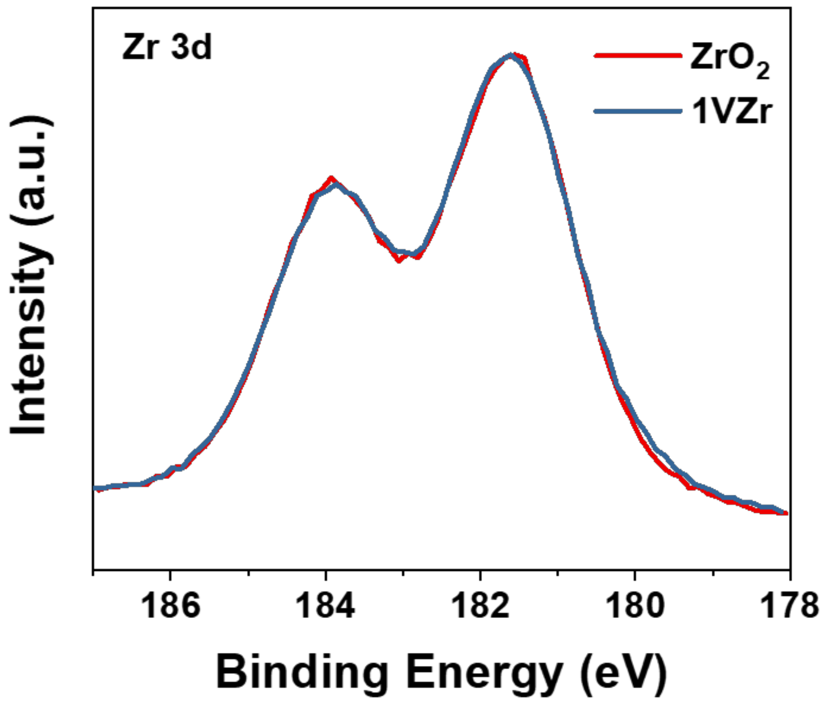

Figure S5. XPS Zr 3d peaks of reduced ZrO_2 and 1VZr.

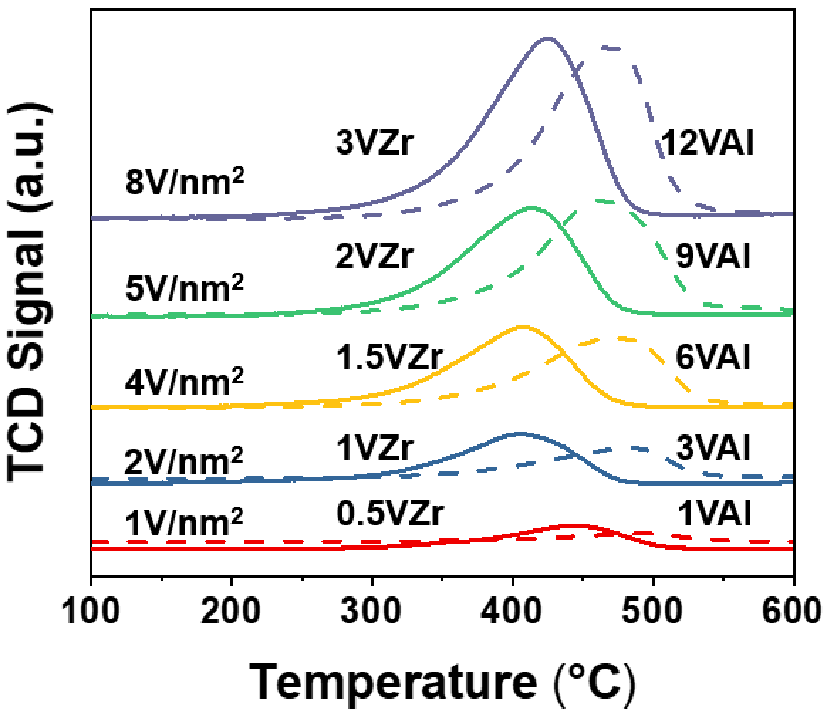

Figure S6. H_2-TPR profiles of VZr and VAl with a series of V loadings. The signal of VZr catalysts are 4 times amplified.

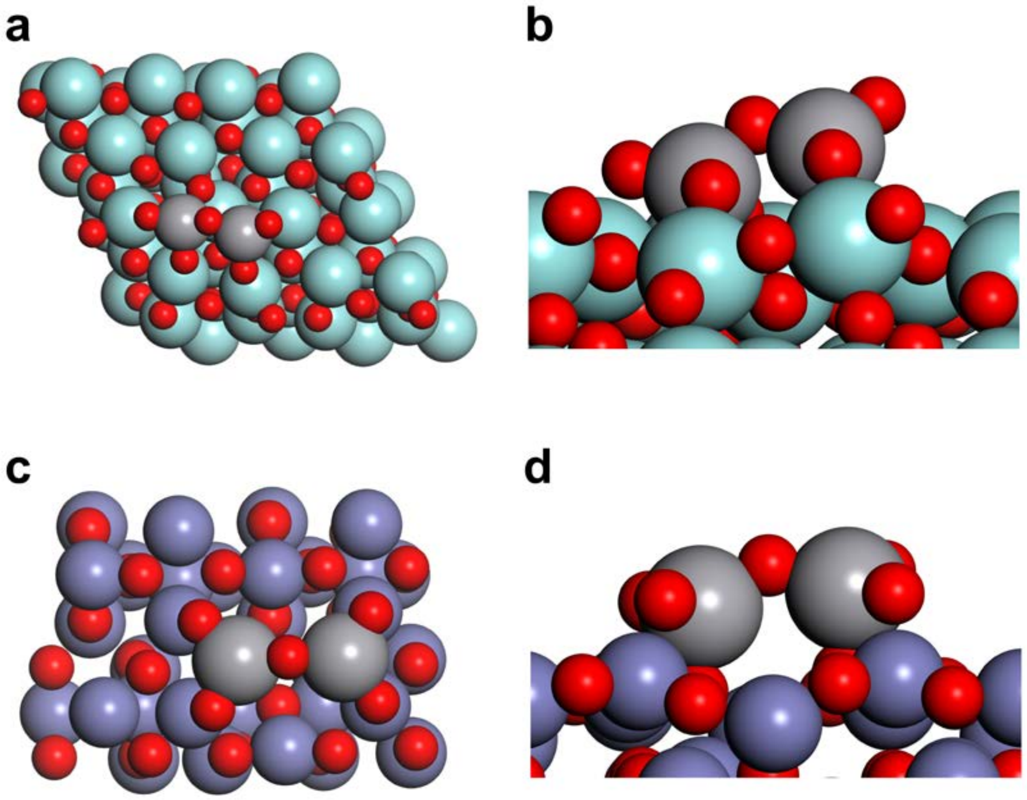

Figure S7. (a) Top- and (b) side-view of dimeric V_2O_5 supported on m-$ZrO_2(\bar{1}11)$. (c) Top- and (d) side-view of dimeric V_2O_5 supported on γ-Al_2O_3(100). Color scheme: V gray; O red; Zr cyan; Al purple.

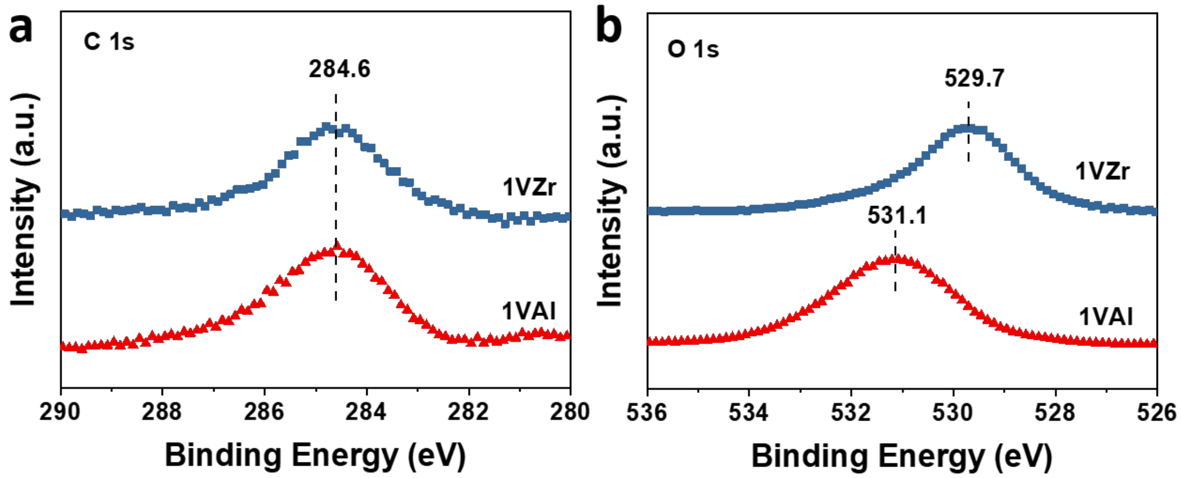

Figure S8. XPS (a) C 1S (b) O 1s peaks of 1VZr and 1VAl after 30 mins reduction.

Reference

1. G. Liu, Z.-J. Zhao, T. Wu, L. Zeng and J. Gong, ACS Catal., 2016, **6**, 5207-5214.
2. U. Rodemerck, M. Stoyanova, E. V. Kondratenko and D. Linke, J. Catal., 2017, **352**, 256-263.
3. U. Rodemerck, S. Sokolov, M. Stoyanova, U. Bentrup, D. Linke and E. V. Kondratenko, J. Catal., 2016, **338**, 174-183.
4. C. A. Carrero, R. Schloegl, I. E. Wachs and R. Schomaecker, ACS Catal., 2014, **4**, 3357-3380.
5. J. T. Grant, C. A. Carrero, A. M. Love, R. Verel and I. Hermans, ACS Catal., 2015, **5**, 5787-5793.
6. P. Hu, W.-Z. Lang, X. Yan, L.-F. Chu and Y.-J. Guo, J. Catal., 2018, **358**, 108-117.
7. G. Kresse and J. Furthmüller, Phys. Rev. B, 1996, **54**, 11169.
8. G. Kresse, J. Non-Crys. Solids, 1995, **192**, 222-229.
9. J. P. Perdew, K. Burke and M. Ernzerhof, Phys. Rev. Lett., 1996, **77**, 3865-3868.
10. P. E. Blöchl, Phys. Rev. B, 1994, **50**, 17953-17979.
11. G. Henkelman, B. P. Uberuaga and H. Jónsson, J. Chem. Phys., 2000, **113**, 9901-9904.
